# Activation and Role of NACHT, LRR, and PYD Domains-Containing Protein 3 Inflammasome in RNA Viral Infection

**DOI:** 10.3389/fimmu.2017.01420

**Published:** 2017-10-31

**Authors:** Junyang Yu, Yuzhang Wu, Jingxue Wang

**Affiliations:** ^1^Institute of Immunology, Third Military Medical University, Chongqing, China

**Keywords:** ribonucleic acid virus, NACHT, LRR, and PYD domains-containing protein 3 inflammasome, activation, effects, viral infection

## Abstract

NACHT, LRR, and PYD domains-containing protein 3 (NLRP3) inflammasome activation and effects during ribonucleic acid (RNA) viral infection are the focus of a wide range of research currently. Both the pathogen-associated molecule pattern derived from virions and intracellular stress molecules involved in the process of viral infection lead to activation of the NLRP3 inflammasome, which in turn triggers inflammatory responses for antiviral defense and tissue healing. However, aberrant activation of the NLRP3 inflammasome can instead support viral pathogenesis and promote disease progression. Here, we summarize and expound upon the recent literature describing the molecular mechanisms underlying the activation and effects of the NLRP3 inflammasome in RNA viral infection to highlight how it provides protection against RNA viral infection.

## Introduction

The ribonucleic acid (RNA) virus has single-stranded (ss)RNA or double-stranded (ds)RNA as its genetic material. Infections with RNA viruses are responsible for a variety of diseases are significant threats to public health, including influenza, hepatitis, viral encephalitis, and autoimmune deficiency syndrome, to name a few of the most prominent. Host antiviral defense mechanisms are critical for clearing RNA viral infections and controlling the manifested diseases. The initiation of antiviral defense in response to RNA viruses involves interferon (IFN) response and inflammasome activation. The RNA viral infection usually leads to activation of the NACHT, LRR, and PYD domains-containing protein 3 (NLRP3) inflammasome, which is the intracellular multiprotein complex formed upon sensing pathogen-associated molecule patterns and/or damage-associated molecule patterns (1). The inflammasome response is critical for the innate immune response’s ability to initiate innate and adaptive immunity pathways during RNA viral infections (2).

The NLRP3 inflammasome has been extensively investigated. Its major components include pro-caspase-1 and apoptosis-associated speck-like protein containing a CARD (ASC), in addition to the NLRP3 molecules. Upon sensing activating signals, the NLRP3 oligomerize to generate a caspase-1-activating scaffold, resulting in autocleavage, and activation of pro-caspase-1. The activated caspase-1 then shears pro-interleukin (IL)-1β and pro-IL-18 into IL-1β and IL-18, leading to production of their functional forms and release from cells to mediate protective (or sometimes detrimental) inflammatory responses. The activated caspase-1, however, can also promote caspase-1-dependent pyroptosis, a type of inflammatory programmed cell death; although, the consequence of pyroptosis induced by the NLRP3 inflammasome remains to be fully elucidated in RNA viral infection (3).

Multiple lines of evidence have shown that the NLRP3 inflammasome can be activated by various viruses, including type A Influenza virus (IAV), human immunodeficiency virus-type 1 (HIV-1), vesicular stomatitis virus (VSV), respiratory syncytial virus (RSV), enterovirus 71 (EV71), hepatitis C virus (HCV), and SARS coronavirus (SARS-CoV), as detailed in Table 1. This review will focus on recent advances in our knowledge of the activation mechanisms and functions of the NLRP3 inflammasome in response to such RNA viral infections, discussing the related protective, and/or detrimental mechanisms that may represent manipulable targets for prevention and/or treatment.

**Table 1:** Activating signals for and roles of the NACHT, LRR, and PYD domains-containing protein 3 (NLRP3) inflammasome in ribonucleic acid (RNA) viral infections.

Virus	Activating signal	Role	Phenotype	Reference
Influenza A	Viroporin M2, PB1-F2, vRNA	Protective detrimental	Mice lacking the NLRP3 inflammasome displayed dramatically increased mortality following IAV challenge. MCC950 treatment from day 3 or 7 after IAV infection protected the mice from severe and highly virulent IAV HKx31-induced disease	(4–9)
HIV	Vpr, Tat	Detrimental	NLRP3 activation in the microglia contributed to HIV-associated neuroinflammation. Podocyte pyroptosis could be involved in the related HIV-associated nephropathy	(10–12)
RSV	SH protein, vRNA	Protective	Overexpression of IL-18 attenuated the peak viral load by three-fold	(13–15)
WNV	?	Protective	In NLRP3^−/−^ and IL-1R^−/−^ mice, disease susceptibility, clinical disease severity, and brain viral load increased following WNV-TX infection	(16, 17)
HCV	Viroporin p7, vRNA	Detrimental	The NLRP3 inflammasome-dependent IL-1β promotes hepatic inflammation. In a human hepatoma cell line, NLRP3-dependent caspase-1 degraded INSIG proteins, thereby inducing the activation of SREBPs and subsequent accumulation of lipid droplets	(18–21)
Dengue virus	?	Detrimental	The NLRP3-dependent IL-1β derived from platelets is associated with the signs of increased vascular permeability	(22)
Sindbis virus	?	Detrimental	IL-1β increases the severity of paralysis during encephalomyelitis induced by the non-virulent strain of Sindbis virus	(23, 24)
SARS-CoV	E protein	Detrimental	Activation of the NLRP3 inflammasome and over-production of IL-1β is associated with pulmonary damage, edema, and death	(25)
ECMV	Viroporin 2B	Protective	Injection with IL-18 from day 1, and not day 2 or 5, after EMCV infection improved the survival rates of mice	(26, 27)
VSV	vRNA	?	Caspase-1-deficient mice are not more susceptible to VSV infection than wild-type mice	(28–30)
Rhinovirus	Viroporin 2B	Detrimental	Blockade of IL-1β by neutralizing antibody decreased viral titers in the supernatants of human tracheal epithelial cells infected by human rhinovirus-14	(31, 32)
EV71	3D protein	Protective	NLRP3^−/−^ mice infected by EV71 showed earlier occurrence of disease, stronger paralysis, delayed recovery, higher morbidity rates, and increased viral load compared with wild-type mice	(33, 34)
Sendai virus	vRNA	Protective	Recombinant IL-1β provides protection against Sendai virus infection in mice	(35, 36)
Zika virus	?	Detrimental	NLRP3 activation in the microglia results in neuroinflammation and cell death	(37)
Coxsackievirus B3	?	Detrimental	The NLRP3 inflammasome is involved in pathogenesis of CVB3-induced myocarditis	(38)
JEV	?	?	NLRP3 inflammasome activation could be associated with neuroinflammatory injury	(39, 40)

*?, unknown currently*.

## Priming Mechanisms of the NLRP3 Inflammasome in RNA Viral Infections

NACHT, LRR, and PYD domains-containing protein 3 inflammasome activation requires two types of signals. Signal 1 primes the NLRP3 inflammasome and signal 2 initiates the sequence of its assembly, activation of the pro-caspase-1, and cleavage of pro-IL-1β and pro-IL-18.

During RNA viral infections, signal 1 is usually derived from the signals evoked upon pathogen recognition by toll-like receptors (TLRs) (6) and retinoic acid-inducible gene-I (RIG-I)-like receptors (41). Recent research has shown that signal 1 triggering of the NLRP3 inflammasome involves transcription-dependent and post-translation-dependent priming. The transcription-dependent priming is executed by activation of nuclear factor (NF)-κB and IFN-regulatory factor transcription factors, and subsequent up-regulation of transcription of genes encoding the major inflammasome components through the classical TLR signaling pathway: the MyD88-mediated and TRIF-mediated pathway. The specific role of TLR7 was shown by the reduced pro-IL-1β transcription in bone marrow-derived macrophages from TLR7^−/−^ mice in response to 24 h of IAV infection (6). Another *in vivo* study of the IAV infection model provided evidence that commensal bacteria lipopolysaccharide can provide priming signal 1 (42). Investigations of VSV infection showed that RIG-I promotes the synthesis of pro-IL-1β through engagement by the viral 5′-triphosphate RNA (3pRNA). Moreover, bone marrow-derived cells lacking RIG-I, as well as the downstream molecules MAVS and CARD9, showed reduced synthesis of pro-IL-1β but normal caspase-1 p10 subunits when stimulated with VSV and 3pRNA (41), suggesting that the RIG-I/MAVS/CARD9 signaling pathway provides the signal 1 in VSV infection.

Post-translation-dependent priming occurs upon the simultaneous engagement of TLRs and Nod-like receptors with their ligands, even causing rapid activation of the NLRP3 inflammasome (within 15 min) (43, 44). Viral RNA (vRNA) molecule-induced NLRP3 inflammasome activation also comes about by post-translational priming, but involving the RIP1/caspase 8/RIP3 signaling pathway (29, 45), which may ultimately result in the deubiquitination of NLRP3 protein by BRCC3 (46, 47). RIP3-deficient mice showed reduced caspase-1 activation and IL-1β production following infection with VSV, Sendai virus, or IAV (29). Bone marrow-derived macrophages and mice lacking RIP1 both displayed substantial reduction in caspase-1 cleavage and levels of IL-1β and IL-18 following challenge with IAV (8, 29). In various RNA viral infection studies, the RIP1/RIP3-mediated signaling pathway has been demonstrated to provide the endogenous signal 2 for the NLRP3 inflammasome, being derived from reactive oxygen species (ROS) production by mitochondrial fission initiated by the RIP1/RIP3/DRP pathway (29, 45, 48).

## Viral Proteins Involved in NLRP3 Inflammasome Activation Induced by RNA Virus

Signal 2 is responsive to a wide range of stimuli [i.e., NLRP3 agonists, such as adenosine triphosphate (ATP), silica, alum, and ultraviolet radiation]. However, the spectrum of different structures represented by these varied and distinctive agonists indicates that NLRP3 does not simply bind directly to each but instead relies on a common intracellular signal transmission pathway, triggered by the agonists themselves, for activation of the NLRP3 inflammasome. To date, five major signal mechanisms have been proposed as those responsible for or contributing to the NLRP3 inflammasome activation (1, 49). In RNA viral infections, in particular, viroporins and some viral functional proteins act as the stimuli for signal 2 (as shown in Table 1).

Viroporins are a group of virus-encoded proteins that enhance permeability of host cell membrane (through interactions with the lipid bilayer) to promote release of viral particles from cells (50), making them a critical component of virus replication and virion release in the host system. The viroporin effect on permeability also modifies the cell’s ability to regulate ion passage, disrupting ion homeostasis (51). Several viroporins, such as the IAV M2 protein (6), RSV SH protein (15), HCV p7 protein (21), SARS-CoV E protein (25), EMCV 2B protein (27), and rhinovirus 2B protein (32), have been reported to activate the NLRP3 inflammasome by disturbing intracellular ionic concentrations, particularly through potassium efflux, calcium flux, and pH alteration. In the viroporin-induced NLRP3 activation itself, the host-encoded NEK7 protein may contribute to the NLRP3 inflammasome assembly and activation *via* formation of the NLRP3-NEK7 complex (52).

Among the other viral proteins involved in NLRP3 inflammasome activation are IAV PB1-F2 (a small protein encoded by an alternate + 1 open reading frame in the viral PB1 gene) (7) and EV71 3D protein (an RNA-dependent RNA polymerase) (34). PB1-F2 activates the NLRP3 inflammasome through mitochondrial ROS production, which could be a major mechanism of pathogenic inflammation induced by highly pathogenic IAV strains (53). The ZBP1 protein can interact with RIP3 upon sensing the viral protein PB1, and contributes to NLRP3 inflammasome activation in IAV but not in VSV infection through post-translational regulation (8). In contrast, the EV71 3D protein can directly bind to NLRP3 and ASC, and promotes NLRP3 inflammasome assembly and activation (34).

## vRNA-Associated Mechanisms Contributing to NLRP3 Inflammasome Activation

Plasma vRNA, another common agonist for NLRP3, may bind with NLRP3 and act to subsequently activate it. Although NLRP3 contains a nucleotide-binding domain (54), RNA-sensing molecules have been reported to participate in the process by which vRNA activates the NLRP3 inflammasome; these include the DExD/H-box helicase (DHX) family members (14, 41, 55, 56) and the 2′,5′-oligoadenylate synthetase (OAS, or 2-5A)/RNase L system (30). However, some debate exists as to the exact role of DHX33, in particular, in VSV-induced NLRP3 inflammasome activation (29).

The DHX family members contain some domains required for ATP binding, hydrolysis, and nucleic acid binding and unwinding, which usually acts as a cytosolic RNA sensor and triggers type I IFN response. DHX33, DDX19A, and DDX58 have been reported to regulate NLRP3 inflammasome activation. DHX33 was shown to combine directly with vRNA to bind to NLRP3 through the DEAD domain of DHX33 and the NACHT domain of NLRP3, forming a DHX33/NLRP3/ASC inflammasome complex to promote NLRP3 inflammasome activation (14). In that same study, down-regulation of DHX33 expression led to a substantial reduction in cleavage of caspase-1 and secretion of IL-18 and IL-1β from the human monocyte cell line, THP-1, upon stimulation with viral dsRNA purified from reovirus, and produced by RSV during the replication process (14). DHX33 has also been demonstrated to contribute to NLRP3 inflammasome activation and IL-1β secretion during VSV and IAV infections (30), the process of which was found to be dependent upon the OAS/RNase L system induced by IFN. OASs recognize cytosolic viral dsRNA and subsequently trigger synthesis of 2–5A in the presence of three ATP molecules. Then, RNase L is activated by 2–5A and cleaves intracellular vRNA. These RNase L-mediated RNA cleavage products will directly interact with DHX33 and induce a DHX33/MAVS/NLRP3 complex to trigger assembly of the NLRP3 inflammasome.

Another study demonstrated that DDX19A binds porcine reproductive and respiration syndrome virus (PRRSV) genomic RNA through its ATP-binding domain and C-terminal domain and directly interacts with the NLRP3 inflammasome in highly pathogenic-PRRSV-infected primary porcine alveolar macrophages, dependent on the DDX19A ATP-binding domain, and the NLRP3 NACHT and LRR domains. Knockdown of DDX19A expression led to reductions in both IL-1β secretion and virus titers in the PRRSV-infected porcine alveolar macrophages (56). Thus, DDX19A appears to be required for both antiviral defense and NLRP3 inflammasome activation. RIG-I (DDX58) is a key intracellular sensor for cytosol ssRNA and provides the transcriptional priming signal for NLRP3 inflammasome during infections with VSV (41) and IAV (55), upon recognition of the vRNA. However, in primary respiratory epithelial cells, IAV infection promotes RIG-I binding with ASC and caspase-1, implying that RIG-I contributes to inflammasome assembly (55).

## Effects Induced by the NLRP3 Inflammasome During RNA Viral Infections

During the process of RNA viral infection, NLRP3 inflammasome activation contributes to either enhancing antiviral defense and tissue healing, or inflammatory pathological injury, which depends mainly on the time point and extent of NLRP3 activation. The appropriate and early phase activation of the NLRP3 inflammasome in some RNA viral infection systems, such those of IAV, encephalomyocarditis virus, and West Nile virus (WNV) (shown in Table 1), usually provide protection, thereby decreasing mortality and viral load, as shown in virus-infected mouse models (4, 9, 16, 57). In rhesus monkeys, neither NLRP3 inflammasome activation nor IFN response can be established successfully within 24 h following challenge with the simian immunodeficiency virus, which may be the key reason underlying the observation of viral dissemination occurring rapidly from the inoculation site to a distal site (within the first day of infection) (58). Reciprocally, when using HIV-1-specific broadly neutralizing antibodies, PGT121 is capable of inducing innate immune responses such as NLRP3 inflammasome activation in vRNA-positive tissues within 24 h; in addition, viral replication is reduced in distal tissues significantly (59). These findings imply the early NLRP3 inflammasome activation as well as IFN response play critical roles in establishment of effective antiviral defense. Similarly, the administration of MC9550 (a specific NLRP3 inhibitor) on days 1, 3, and 5 but not days 3–5 following IAV challenge also resulted in accelerated weight loss and mortality in IAV-infected mice (9). At 2 weeks after challenge with IAV, the mortality rates among NLRP3^−/−^, caspase-1^−/−^ (4, 5), and IL-1RI^−/−^ (57) mice are significantly higher (by 60–80%) than for the wild-type counterparts. Thus, proper NLRP3 inflammasome activation in the early phase of RNA viral infection protects the infected host. In contrast, if activated at a later phase or over-activated, the NLRP3 inflammasome response to RNA virus results in inflammatory injury.

**Figure 1:**
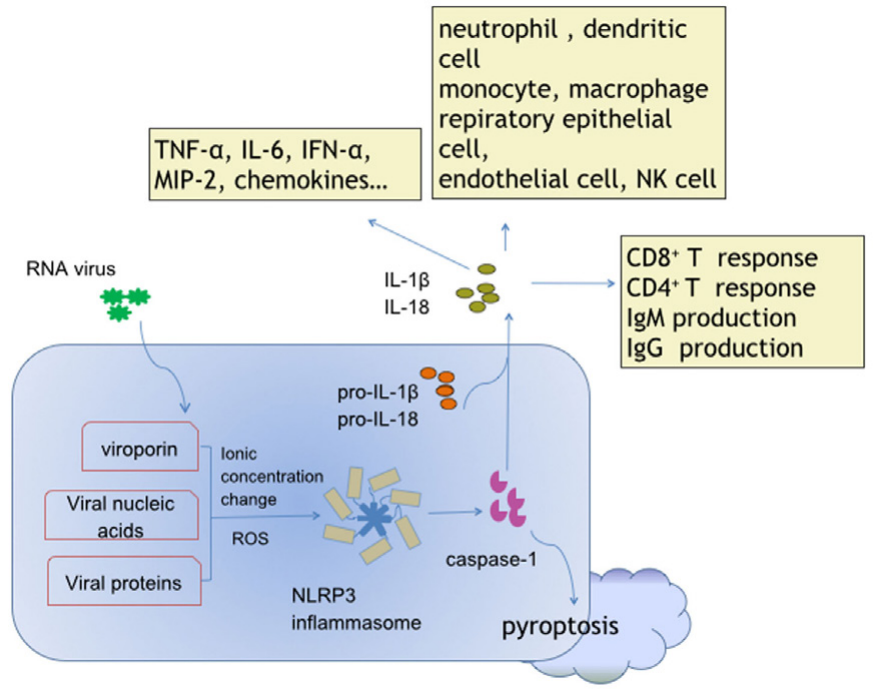
Activation and effects of NACHT, LRR, and PYD domains-containing protein 3 (NLRP3) inflammasome in response to ribonucleic acid (RNA) virus infections. Viroporin, viral nucleic acids, and some virus-encoded functional proteins are able to activate the NLRP3 inflammasome. Viroporin-mediated ion concentration change, virus replication-produced reactive oxygen species, and even viral protein direct binding promote assembly of the NLRP3 inflammasome. The cytokines interleukin (IL)-1 and IL-18 contain extensive downstream effector molecules and effector cells, which ultimately orchestrate innate and adaptive immunity, leading to antiviral defense, and/or inflammatory injury during RNA virus infections.

Production of IL-1β and IL-18, and pyroptosis are the critical and fundamental reactions directly induced by activated NLRP3 inflammasome (Figure 1). IL-1β and IL-18 serve to activate myriad downstream cell responses, and orchestrate innate and adaptive immunity through MyD88/IRAK4/TRAF6-mediated NF-κB signaling and the JNK/p38 mitogen-activated protein kinase pathways (60–63), which may represent key events for the NLRP3 inflammasome-dependent antiviral defense. Pyroptosis is a pro-inflammatory caspase-dependent programed cell death (64). NLRP3 inflammasome-dependent pyroptosis has been described in HCV-infected hepatoma cells (65), Dengue virus (DV)-infected monocytes (66), and HIV-infected podocytes (10). The data to date suggest that pyroptosis in viral infection leads to host injury and pathogenesis.

In mice, IAV-induced NLRP3 inflammasome activation initiates protective inflammation by recruitment of monocytes and neutrophils to the lung (as noted in the bronchoalveolar lavage fluid), which occurs within 3–7 days of the IAV infection, and the subsequent promotion of secretion of various cytokines, such as tumor necrosis factor (TNF)-α, IFN-α, and MIP-2α (4, 5). Furthermore, the impaired IL-1 response by IL-1RI deficiency results in impaired production of immunoglobulin M, reduced recruitment of CD4^+^ T cells in the bronchoalveolar lavage fluid (57), and impaired priming of CD8^+^ T cells in the lung and draining mesenteric lymph nodes through the intervention of CD11b^lo^CD103^+^ dendritic cell migration and maturation (67). In a recent study, IL-18 was confirmed to be required for IFN-γ production in CD161^+^Vα7.2^+^ mucosal-associated invariant T cells, which has been observed clinically to be increased in recovered but not succumbed patients hospitalized with avian H7N9 influenza pneumonia and which implies a protective role in influenza virus infection (68). In WNV infected mice, the IL-1β produced by CD45^+^CD11b^+^ infiltrating T cells promotes the WNV-specific CD8^+^ T cell entry to the central neuronal system *via* regulation of CXCL12-mediated T cell adhesion, eventually controlling the viral infection (16, 17). In BV-2 mouse microglia cells infected by Japanese encephalitis virus, the NLRP3 inflammasome induces production of IL-1β and IL-18 rapidly (within 3 h of exposure) and of TNF-α, CCL2, and IL-6 later (within 6 h after exposure) (40); the findings suggest that the NLRP3-dependent protective inflammatory response is a very early phase innate immune response against RNA viral infection. In addition to initiating protective inflammation to regulate and orchestrate innate and adaptive immunity, the NLRP3 inflammasome also promotes tissue healing and reduces respiratory epithelial necrosis, which contributes to enhancing host disease tolerance (5, 69). Thus, the NLRP3 inflammasome provides protective roles through enhancing host tolerance capacity and orchestrating innate immunity and adaptive immunity during RNA viral infections.

Aberrant NLRP3 inflammasome activation usually results in progression of viral pathogenesis and the manifested disease in host, such as the robust NLRP3 activation induced by PB1-F2 during the IAV infection (7). The major pathogenic mechanism associated with PB1-F2-induced aberrant NLRP3 inflammasome activation involves excessive neutrophil influx (7, 53). The NLRP3 activation induced by HCV promotes chronic intrahepatic inflammation in macrophages (18) and interrupts cellular lipid metabolism (20) in HCV-infected hepatoma cells, both of which contribute to chronic liver injury and liver disease progression. In HIV-infected microglia, NLRP3 inflammasome activation is involved in the occurrence of chronic neuroinflammation (12). In addition, NLRP3-dependent pyroptosis during RNA viral infection also supports the pathogenesis of virus infection diseases. HIV-induced podocyte pyroptosis could be associated with HIV-associated nephropathy (10). NLRP3 inflammasome-mediated pyroptosis is also observed in DV-infected monocytes (66) and in HCV-infected and bystander hepatoma cells (65), and could be associated with viral pathogenesis (Table 1).

## Negative Regulation of NLRP3 Inflammasome by RNA Virus

Unlike RNA viruses, the DNA viruses, which have a large DNA genome, can encode viral homologs of some complex intracellular inflammasome-regulating molecules to regulate inflammasome activity; an example of this is the pyrin-only proteins and BCL-2 encoded by poxvirus (70, 71). The RNA viruses, in contrast, have evolved some simple yet effective strategies to inhibit activation of the NLRP3 inflammasome and evade host immunity. Specifically, the proteases 3C and 2A of EV71 are able to cleave NLRP3 at the G493-L494 or Q225-G226 junction (34). Furthermore, the protease 3C of EV71 is capable of degrading gasdermin D, a protein critical to the induction of pyroptosis (72), and host failure to inhibit EV71 replication (73). The NS1 protein of influenza virus can bind with NLRP3 directly, thereby inhibiting assembly of the NLRP3-ASC-caspase-1 complex and secretion of IL-1β; this inhibition is dependent on RNA and the TRIM-25 domain of the NS1 protein (74). The measles virus V protein also targets NLRP3, probably serving to interfere with the NLRP3 inflammasome assembly and ultimately facilitating escape from the host immune response (75). Finally, the non-structural protein 11 encoded by PRRSV also antagonizes the NLRP3 inflammasome effect in the macrophages, through decreasing expression of pro-IL-1β (76).

## Concluding Remarks

NACHT, LRR, and PYD domains-containing protein 3 inflammasome activation in response to RNA virus is a fundamental innate immune response as well as an IFN response, which usually contributes to antivirus defense, although aberrant and inappropriate NLRP3 inflammasome response still results in the immunopathology and exaggeration of infectious disease, especially in some persistent virus infections. The activation itself is induced by different signals and probably occurs in different phases of the viral infection, triggering inflammatory response (appropriate/protective or inappropriate/detrimental), and affecting outcome of the viral infection substantially. Thus, the proper regulation of NLRP3 inflammasome activation with specific NLRP3 inhibitors may have therapeutic benefit in controlling virus-related diseases and in clearance of the infecting virus.

## Author Contributions

JY collecting literatures and drafting manuscript. JW design of the work, collecting literatures, drafting, and revising manuscript. YW discussing literatures, design of the work, and revising manuscript.

## Conflict of Interest Statement

The authors declare that the research was conducted in the absence of any commercial or financial relationships that could be construed as a potential conflict of interest.
